# Tricuspid Annular Plane of Systolic Excursion (TAPSE) for the Evaluation of Patients with Severe Sepsis and Septic Shock

**DOI:** 10.5811/westjem.2019.11.44968

**Published:** 2020-01-13

**Authors:** Shadi Lahham, Clifton Lee, Qumber Ali, John Moeller, Chanel Fischetti, Maxwell Thompson, Soheil Saadat, John C. Fox

**Affiliations:** * University of California, Irvine; Department of Emergency Medicine, Orange, California; † University of Alabama, Department of Emergency Medicine, Birmingham, Alabama

## Abstract

**Introduction:**

Sepsis is a systemic infection that can rapidly progress into multi organ failure and shock if left untreated. Previous studies have demonstrated the utility of point of care ultrasound (POCUS) in the evaluation of patients with sepsis. However, limited data exists on the evaluation of the tricuspid annular plane of systolic excursion (TAPSE) in patients with sepsis.

**Methods:**

We prospectively enrolled patients who presented to the emergency department (ED) with concern for severe sepsis or septic shock in a pilot study. In patients that screened positive, the treating physician then performed POCUS to measure the TAPSE value. We compared the intensive care unit (ICU) admission rate, hospital length of stay, and morbidity with their respective TAPSE values.

**Results:**

We enrolled 24 patients in the study. Eight patients had TAPSE values less than 16 millimeters (mm), two patients had TAPSE values between 16mm–20mm, and fourteen patients had TAPSE values greater than 20mm. There was no statistically significant association between TAPSE levels and ICU admission (p=0.16), or death (p=0.14). The difference of length of stay (LOS) was not statistically significant in case of hospital LOS (p= 0.72) or ICU LOS.

**Conclusion:**

Our pilot data did not demonstrate a correlation between severe sepsis or septic shock and TAPSE values. This may be due to several factors including patient comorbidities, strict definitions of sepsis and septic shock, as well as the absence of septic cardiomyopathy (SCM) in patients with sepsis and septic shock. Future large-scale studies are needed to determine if TAPSE can be beneficial in the ED evaluation of patients with concern for SCM.

## INTRODUCTION

Sepsis is a systemic infection that can rapidly progress into multi organ failure and shock if left untreated. As a disease process, sepsis carries up to a 25% mortality rate and affects millions of patients annually.^1^ Most commonly, a bacterial infection causes a systemic inflammatory cascade leading to a spectrum of physiologic changes.^2^,^3^ The sequelae of sepsis can lead to significant morbidity and mortality in the setting of delayed treatment. As such, there has been increasing pressure on emergency departments (ED) in the United States to rapidly initiate antibiotics and resuscitative care to patients in early stages of the disease process. Currently, patients are screened using vital sign abnormalities, basic laboratory data, and clinical gestalt. Patients with presumed sepsis are often empirically resuscitated with intravenous fluids and broad-spectrum antibiotics.^4^,^5^

Septic cardiomyopathy (SCM) is defined as a reversible cardiac dysfunction that results in decreased ejection fraction.^6^ In patients with sepsis, the presence of septic cardiomyopathy (SCM) is known to result in significant negative clinical outcomes and a three-to four-fold increase in mortality.^6^ Despite the success of sepsis algorithms, a standardized treatment (such as a 30 cubic centimeters per kilogram [cc/kg] fluid bolus) may have uniquely negative consequences in patients with pre-existing conditions such as SCM, congestive heart failure, or pulmonary hypertension. SCM occurs via several different mechanisms. This includes tachycardia, hypotension and eventual end organ damage.^7^ While most literature regarding the prognostic implications of SCM has focused on left ventricular function, few studies have evaluated the association between right ventricular dysfunction and sepsis.^8^,^9^ Furthermore, accurately studying the effects of sepsis on SCM is a challenging task due a broad range of variables including systolic vs diastolic ventricular impairment, cardiac output, and end-organ tissue injury.^10^ The characterization of sepsis in SCM and alternative management strategies has great potential to positively impact morbidity and mortality in a population predisposed to poor outcomes in the setting of pre-existing conditions.

Previous studies have demonstrated the utility of point of care ultrasound (POCUS) in the evaluation of patients with sepsis.^11^,^12^ However, limited data exists on the evaluation of POCUS for septic cardiomyopathy. There are no specific or sensitive sonographic signs for identifying sepsis or septic cardiomyopathy (SCM) other than those associated with infection at a specific site. Most literature describes SCM in terms of a relationship to left ventricular dysfunction.^13^ However, measurements of left ventricular function do not explain the relationship between the high preload from resuscitative efforts. Our study aims to assess the right ventricle in patients with severe sepsis. Specifically, we aim to evaluate tricuspid annular plane systolic excursion (TAPSE), which is an effective indirect method of evaluating right ventricular (RV) function.^14^ The American Society of Echocardiography recommends the use of TAPSE as a quantitative method of estimating RV function. Additionally, previous studies have demonstrated that emergency physicians (EPs) are capable of obtaining TAPSE measurements in ED patients.^15^,^16^

The objective of this pilot study is to evaluate the relationship between RV dysfunction as measured by POCUS obtained TAPSE values in patients presenting to the ED with concern for severe sepsis and septic shock.

**Population Health Research Capsule**

What do we already know about this issue?

Sepsis is a systemic infection that can rapidly progress if left untreated. No studies have evaluated the tricuspid annular plane of systolic excursion (TAPSE) in patients with sepsis.

What was the research question?

Can TAPSE values be useful in evaluating patients with sepsis and septic shock?

What was the major finding of the study?

Our pilot data did not demonstrate a correlation between severe sepsis or septic shock and TAPSE values.

How does this improve population health?

Point-of-care ultrasound is useful in evaluation of patients with sepsis. However, TAPSE may not be predictive of outcomes for patients with sepsis or septic shock.

## MATERIALS AND METHODS

### Study Design and Settings

We performed a prospective, observational single-site pilot study using a convenience sample of patients who presented to the ED between March 2018 and February 2019. Patients were enrolled in an urban university hospital ED, which supports an emergency medicine (EM) residency training program as well as an EM ultrasound fellowship. The annual ED census consists of approximately 57,000 patient visits annually with an ethnically and economically diverse patient population. The study was approved by the University of California institutional review board and follows the Standards for Reporting of Diagnostic Accuracy Studies guidelines.

### Selection of Participants

Research associates monitored the ED track board for potential patients daily between the hours of 8:00 am and 12:00 midnight. Patients were eligible for inclusion if they were at least 18 years old, able to provide written and verbal consent in English or Spanish, and were undergoing evaluation for sepsis and septic shock. All laboratory tests and imaging studies were performed at the discretion of the treating physician. Patients were excluded if they were pregnant, incarcerated, mechanically ventilated prior to initial evaluation, unable to provide medical consent, or did not meet inclusion criteria. Patients were also excluded if they had a history of pulmonary hypertension, known pulmonary embolism, or heart failure. The research associates obtained informed and written consent from eligible patients after discussion of the study with the treating physician.

### Study Protocol

In patients that screened positive for severe sepsis or septic shock, the treating physician approached the patient for enrollment in the study. Screening for patients was based on the Third International Consensus Definitions for Sepsis and Septic Shock, which includes fever, tachycardia, and hypotension. Screening criteria included at least two of the following: temperature > 38 C or < 36 C, heart rate > 90 beats per minute, respiratory rate > 20 breaths per minute or partial pressure of carbon dioxide (PaCO2) < 32 millimeters of mercury (mmHg), white blood cell (WBC) count > 12,000/mm^3^, < 4,000/mm^3^, or > 10% bands, and a suspected or present source of infection. For patients with severe sepsis or septic shock, additional criteria included hypotension despite adequate fluid resuscitation or evidence of ≥ 2 organs failing.^5^

Any patient that met criteria was approached for enrollment in the study. Following verbal and written consent, the research team collected data using a systematic approach on a standard data abstraction sheet. Collected data included general demographics such as age and gender, along with history of heart failure, pulmonary hypertension, pulmonary embolism, chronic obstructive pulmonary disease, hypertension, and smoking. Initial vital signs were also collected, along with POCUS measurements in real time during evaluation. Following enrollment and treatment, retrospective data was collected including length of hospital stay (LOS), intensive care unit (ICU) admittance, incidence of respiratory failure, and/or mortality.

### TAPSE Protocol

Following consent, the treating emergency physician then performed POCUS to measure the TAPSE value prior to obtaining any laboratory results, imaging test results, or treatment. TAPSE values were obtained using Mindray TE7 (Mindray North America, Mahwah, NJ) ultrasound machines with a phased array transducer in the cardiac software setting. All patients were placed in the left lateral decubitus position to properly obtain an apical 4-chamber view of the heart. An M-mode sampling spike was placed at the right lateral border of the heart at the tricuspid valve annulus, which generated simultaneous live B and M-mode active tracings. A TAPSE value was obtained by measuring the vertical height between the peak and trough in a single cardiac cycle to determine the apex to base shortening.^17^ Patients were then differentiated into three groups. Groups included TAPSE values less than 16 mm, TAPSE 16 mm–20 mm, and TAPSE >20 mm.

A total of 14 unique physicians collected TAPSE measurements. This included EM attending physicians, resident physicians, and emergency medicine ultrasound fellows. Prior to the enrollment of patients in the study, all EM physicians underwent a 30-minute didactic lecture followed by supervised hands-on scanning of three healthy volunteer adult models. All practitioners were required to demonstrate the ability to obtain an apical 4-chamber view and correctly take a TAPSE measurement on three models prior to enrolling patients. All POCUS images were archived and reviewed by the ED ultrasound director to confirm appropriate image quality and accurate measurements.

### Statistical analysis

Frequencies are represented as count (%) and continuous variables as mean ± standard deviation (SD). Chi square test for trend was used to examine the distribution of death and ICU admission per TAPSE levels. We compared the hospital length of stay and the ICU length of stay with their respective TAPSE values using the Kruskal-Wallis test. The distribution of TAPSE value was examined by using the Kolmogorov-Smirnov statistical test. IBM SPSS Statistics for Windows version 25 was used for data analysis.

## RESULTS

32 patients were approached for enrollment in the study. Eight patients were excluded from the final data analysis: two patients declined to participate, five patients reported a history of heart failure, and one patient had a history of pulmonary embolism and was on anticoagulation. A total of 24 patients were enrolled in the study. Four patients (16.7%) were female and 20 patients (83.3%) were male. The mean age of the enrolled patients was 56 ± 18. See Table 1 for full patient characteristics.

Patients were organized into three different TAPSE groups. Eight patients had TAPSE values less than 16 mm, two patients had TAPSE values between 16 mm–20 mm, and fourteen patients had TAPSE values greater than 20 mm. The distribution of TAPSE value was not far from normal (P=0.20). The mean TASPE value was 20.8 with SD of 6.68 (Range: 9.6–34.2). In the TAPSE group less than 16 mm, two patients (25%) were admitted to the ICU and none had mortality during admission. In the TAPSE group 16mm–20mm, one (50%) was admitted to the ICU and none had mortality during admission. In the TAPSE group greater than 20mm, 11 (45.8%) were admitted to the ICU and three (21.4%) had mortality during admission. There was not a statistically significant association between TAPSE levels and ICU admission (p=0.16) or death (p=0.14).

The average hospital length of stay (LOS) for each group was 99±51, 184±92 and 132±57 hours respectively. The average ICU LOS for each group was 34±49, 96±48 and 51±38 hours respectively. The difference of LOS was not statistically significant neither in case of hospital LOS (p= 0.72) nor ICU LOS (p=0.75).

## DISCUSSION

Severe sepsis and septic shock are commonly evaluated and treated in the ED. However, currently there are no gold standard ultrasound findings that can be used to identify severe sepsis or SCM. Our study aims to determine if there is a role for the assessment of TAPSE in patients with severe sepsis and septic shock. Our pilot data does not demonstrate any significant difference between ICU admission or mortality based on ED measured TAPSE values. On retrospective review of physician charting, all patients received a resuscitative fluid bolus in addition to antibiotics. Based on previous studies, we divided our patients into three categories (TAPSE value 16 mm or less, TAPSE value 16 mm to 20 mm and TAPSE value greater than 20mm). Although we excluded patients with known heart failure and other cardiac co-morbidities, other factors including age may also play a role in TAPSE values distinct from the effects of severe sepsis or septic shock. The lack of gold standard radiologic findings specific to sepsis combined with the broad definition of sepsis made establishing TAPSE cutoffs difficult. Additionally, altering the measurement cutoffs for RV dysfunction in our study did not yield statistically significant results.

A previous study by Daley et al. evaluated TAPSE values in patients with pulmonary embolism and used a cutoff of 20 mm to yield a 72% sensitivity for detecting pulmonary embolism.^15^ Other literature uses 17 mm as a threshold for right ventricular dysfunction (RVD).^18^ As such, there is no consensus on what TAPSE value predicts worsening or improving right ventricular function or precludes its utility in clinical decision making when evaluating patients with severe sepsis or septic shock. To our knowledge, no previous studies have evaluated the relationship between sepsis or septic cardiomyopathy and TAPSE values. Thus, there are no defined numerical values where TAPSE becomes clinically significant in patients with severe sepsis, septic shock, or SCM.

The most challenging aspect of the research in defining the role of TAPSE in SCM was defining a patient population with sepsis based on non-specific markers, such as vitals, basic laboratory data, and clinical judgement. Recent efforts have been focused on eliminating the Systemic Inflammatory Response Syndrome (SIRS) requirement, as fever, tachycardia, blood pressure, and white blood cell count are too broad to be applied to critically ill patients.^19^ Using standard SIRS as criteria, we captured a broad range of infectious sources as well as a range disease pathogenicity. Additionally, we were challenged in attempting to control for the numerous comorbidities with known associations that impact sepsis and shock.^20^ Other definitions of sepsis including the quick sequential organ failure assessment (qSOFA) was also considered. However, neither definition is narrow enough. This broad definition of sepsis leads to understanding the condition as a spectrum of disease. Recruiting patients for our study also proved difficult due to comorbidities in this patient population. Eight patients (25%) were excluded from data analysis due to existing cardiovascular conditions that may have affected their TAPSE measurement.

Furthermore, patients who meet SIRS criteria and are ultimately diagnosed with severe sepsis or even septic shock may not exhibit septic cardiomyopathy. While using standards to identify and quickly evaluate patients with infection is useful, the correlation between defined sepsis and SCM is unclear and warrants future projects. The evaluation of the right ventricle in an otherwise healthy patient with severe sepsis or septic shock may not demonstrate signs of SCM based on anatomy and physiology. Furthermore, additional values traditionally evaluated in patients with SCM may not always correlate with TAPSE values. This includes lactic acidosis and troponin.^21^ Further studies are warranted to assess the value of TAPSE measurements in select patient populations such as sepsis or SCM. Based on our pilot data, future large-scale studies are needed to evaluate right heart findings in comparison to global cardiac dysfunction in patients with confirmed SCM to better understand the role of TAPSE in this patient population.

## LIMITATIONS

This study has several limitations. A small number of patients (24) were enrolled utilizing a convenience sample population. This may have introduced selection bias and decreased validity. A single site was used, and the findings from this site may not be generalizable to other patient populations. SIRS criteria was used to identify patients with sepsis and septic shock. The variability of sepsis and septic shock allows a broad category of patients to be diagnosed with such conditions making it difficult to study the immediate relationship between TAPSE value and outcomes for these patients. Our study did not seek to determine the amount of training required for proficiency in obtaining or interpreting TAPSE values. Measurements may be affected by operator experience leading to a greater impact on a study, especially with a smaller sample size. Interrater reliability was not measured in this study.

## CONCLUSIONS

Our pilot data did not demonstrate a correlation between severe sepsis or septic shock and TAPSE values. This may be due to several factors including patient comorbidities, strict definitions of sepsis and septic shock, as well as the absence of SCM in patients with sepsis and septic shock. Future large-scale studies are needed to determine if TAPSE can be beneficial in the ED evaluation of patients with concern for SCM.

## Figures and Tables

**Table 1:** Characteristics of study sample.

		Count (%)
Gender	Female	4 (16.7%)
	Male	20 (83.3%)
HTN	No	14 (58.3%)
	Yes	10 (41.7%)
Smoking	No	16 (66.7%)
	Yes	8 (33.3%)
3+ SIRS criteria	No	12 (52.2%)
	Yes	11 (47.8%)
ICU admission	No	10 (41.6%)
	Yes	14 (58.3%)
Mortality	No	21 (87.5%)
	Yes	3 (12.5%)
Age	56±18	Range: 19–87; Median=60
Hospital LOS	118±74	Range: 0–312; Median=12
ICU LOS	38±48	Range: 0–144; Median=24

*HTN*, hypertension; *SIRS*, systemic inflammatory response syndrome; *ICU*, intensive care unit; *LOS*, length of stay.

## References

[1] Singer M, Deutschman CS, Seymour CW (2016). The Third International Consensus Definitions for Sepsis and Septic Shock (Sepsis-3). JAMA.

[2] Lever A, Mackenzie I (2007). Sepsis: definition, epidemiology, and diagnosis. BMJ.

[3] Pulido JN, Afessa B, Masaki M (2012). Clinical spectrum, frequency, and significance of myocardial dysfunction in severe sepsis and septic shock. Mayo Clin Proc.

[4] Rhodes A, Evans LE, Alhazzani W (2017). Surviving Sepsis Campaign: International Guidelines for Management of Sepsis and Septic Shock: 2016. Intensive Care Med.

[5] Dellinger RP, Levy MM, Rhodes A (2013). Surviving sepsis campaign: international guidelines for management of severe sepsis and septic shock: 2012. Crit Care Med.

[6] Vieillard-Baron A (2011). Septic cardiomyopathy. Ann Intensive Care.

[7] Sato R, Nasu M (2015). A review of sepsis-induced cardiomyopathy. J Intensive Care.

[8] Vallabhajosyula S, Kumar M, Pandompatam G (2017). Prognostic impact of isolated right ventricular dysfunction in sepsis and septic shock: an 8-year historical cohort study. Ann Intensive Care.

[9] Romero-Bermejo FJ, Ruiz-Bailen M, Gil-Cebrian J (2011). Sepsis-induced cardiomyopathy. Curr Cardiol Rev.

[10] Beesley SJ, Weber G, Sarge T (2018). Septic Cardiomyopathy. Crit Care Med.

[11] Alonso JV, Turpie J, Farhad I (2019). Protocols for Point-of-Care-Ultrasound (POCUS) in a Patient with Sepsis; An Algorithmic Approach. Bull Emerg Trauma.

[12] Cortellaro F, Ferrari L, Molteni F (2017). Accuracy of point of care ultrasound to identify the source of infection in septic patients: a prospective study. Intern Emerg Med.

[13] Muller-Werdan U, Buerke M, Ebelt H (2006). Septic cardiomyopathy - A not yet discovered cardiomyopathy?. Exp Clin Cardiol.

[14] Portnoy SG, Rudski LG (2015). Echocardiographic evaluation of the right ventricle: a 2014 perspective. Curr Cardiol Rep.

[15] Daley J, Grotberg J, Pare J (2017). Emergency physician performed tricuspid annular plane systolic excursion in the evaluation of suspected pulmonary embolism. Am J Emerg Med.

[16] Lahham S, Fox JC, Thompson M (2019). Tricuspid annular plane of systolic excursion to prognosticate acute pulmonary symptomatic embolism (TAPSEPAPSE study). J Ultrasound Med.

[17] Ueti OM, Camargo EE, de Ueti AA (2002). Assessment of right ventricular function with Doppler echocardiographic indices derived from tricuspid annular motion: comparison with radionuclide angiography. Heart.

[18] Peacock AJ, Naeije R, Rubin LJ (2016). Pulmonary circulation: diseases and their treatment.

[19] Hotchkiss RS, Moldawer LL, Opal SM, Reinhart K, Turnbull IR, Vincent JL (2016). Sepsis and septic shock. Nat Rev Dis Primers.

[20] Sinapidis D, Kosmas V, Vittoros V (2018). Progression into sepsis: an individualized process varying by the interaction of comorbidities with the underlying infection. BMC Infect Dis.

[21] Zochios V, Valchanov K (2015). Raised cardiac troponin in intensive care patients with sepsis, in the absence of angiographically documented coronary artery disease: A systematic review. J Intensive Care Soc.

